# Inclusion complexes of squalene with beta-cyclodextrin and methyl-beta-cyclodextrin: preparation and characterization

**DOI:** 10.55730/1300-0527.3537

**Published:** 2022-12-29

**Authors:** Anh Van NGUYEN, Victor I. DEINEKA, Anh Thi Ngoc VU, Tuan Dinh LE, Hieu TRAN-TRUNG, Tien A. NGUYEN

**Affiliations:** 1 Faculty of Food Science and Technology, Ho Chi Minh City University of Food Industry, Ho Chi Minh City, Vietnam; 2 Institute of Engineering Technologies and Natural Sciences, Belgorod National Research University, Belgorod, Russia; 3 Environmental Analysis Laboratory, Southern Branch of Vietnam-Russia Tropical Center, Ho Chi Minh City, Vietnam; 4 Department of Chemistry, Hanoi Pedagogical University 2, Vinh Phuc, Vietnam; 5 Department of Chemistry, College of Education, Vinh University, Vinh City, Nghe An, Vietnam; 6 Ho Chi Minh City University of Education, Ho Chi Minh City, Vietnam

**Keywords:** Squalene, inclusion complex, β-cyclodextrin, methyl-β-cyclodextrin

## Abstract

The present work aimed to investigate inclusion complexes of squalene with various cyclodextrins (native β-cyclodextrin and methyl-β-cyclodextrin). The production of squalene-β-cyclodextrin inclusion complex was obtained using Response Surface Methodology and obtained inclusion complexes were studied with FTIR spectroscopy, X-ray diffractometry, thermal analysis, and ^1^H-NMR spectrometry. At the same time, squalene content was determined by reversed-phase high-performance liquid chromatography. All results confirmed that squalene was successfully involved in the cyclodextrin cavities. Optimizing the condition in preparation for the squalen-β-cyclodextrin inclusion complex yielded 54.3% with squalene content of 9.01%. The essential difference for the inclusion complex of squalene with methylated beta-cyclodextrin was that no precipitate formed in the initial mixture, and the complex was more efficiently dispersed in water. The conclusions of the inclusion complex formation were confirmed by computer simulation by optimizing the complex geometry using the DFT, MM2, and MP3 methods.

## 1. Introduction

Success in the research direction of bioactive substances is discovering and creating new biologically active substances and optimizing their use. Squalene (2,6,10,15,19,23-hexamethyltetracose-2,6,10,14,18,22-hexaene) is a structurally unique triterpene compound, which is one of the main components (about 13%) of surface lipids skin [1]. From a pharmaceutical view, squalene possesses unique properties with pharmacological, cosmetic, and nutritional potential [2,3]. Squalene reduces skin damage from ultraviolet radiation [4, 5], prevents cardiovascular diseases [6], and has antitumor effects in ovarian, breast, lung, and colon cancers [7, 8]. In nature, sources with rich squalene content were found in the liver of a shark and some seed oils, such as amaranth. As a nonpolar lipid, squalene is not soluble in water, complicating its use with water-soluble compounds.

Beta-cyclodextrins (βCD), both native and modified, consisting of seven glucose units, represent a unique ability to form inclusion complexes with various organic substances by molecular encapsulation known as hostess-guest complexes [9]. Due to the high hydrophilic outer surfaces, these complexes are readily soluble or dispersible in water. Therefore, cyclodextrins and their derivatives became a convenient tool for creating new drugs to improve their solubility, physical properties, and chemical stability by protecting them from oxidation when exposed to daylight [10–12]. However, the solubility of native βCD in water is significantly lower than that of acyclic saccharides because of strong interactions between cyclodextrin molecules inside the crystal lattice. Besides, intramolecular hydrogen bonds appear between hydroxyl groups of native βCD, disrupting the formation of hydrogen bonds with the surrounding water molecules, manifested in their low solubility [13]. Therefore, any modification of the hydroxyl groups in cyclic glycose, even with hydrophobic substituents, leads to a significant increase in water solubility.

In the present work, we present the synthesis of water-dispersible inclusion complexes of squalene with βCD and methyl-beta-cyclodextrin (Me- βCD) and their characterizations studied by a set method. The optimal conditions of the complex formation were deduced using Response Surface Methodology.

## 2. Material and method

### 2.1. Materials

**Figure 1:**
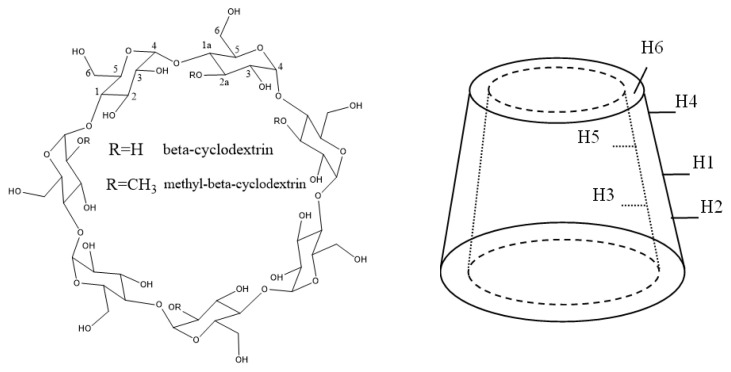
Schematic representation of the beta-cyclodextrin and methyl-beta-cyclodextrin (Me-βCD, Crysmeb).

β-cyclodextrin (βCD, Kleptose®) and randomly methylated β-cyclodextrin (Me-βCD, Crysmeb) were purchased from Roquette Pharma (Lestrem, France), Figure 1. Squalene (98%) was obtained from Alfa Aesar™ (density = 0.858 g/mL).

### 2.2. Preparation of inclusion complex

The inclusion complex was prepared by homogenizing aqueous βCD and Me-βCD (10 mM) solutions after adding a defined squalene volume using a homogenizer (US-4102 Ulab 25000 gm). After that, the mixture was stirred lightly for 2 h for the equilibration of the complexation process.

In the case of the squalene-βCD inclusion complex, the precipitate was separated by centrifugation, washed with 3 mL of 70% ethanol solution three times, and dried on a freeze dryer (Freezone 2.5 Labconco at 0.021 mbar and −48 °C).

The excess squalene was separated for the squalene-Me-βCD complex using the separatory funnel. For this purpose, obtained emulsion solution was delivered into a separatory funnel, then 20 mL n-hexane was added and stirred. The inclusion complex emulsion in the bottom layer was separated. The water-dispersible inclusion complex solution was dried using the freezing method.

### 2.3. HPLC analysis for analyzing squalene content

Squalene was analyzed by reversed-phase HPLC. Chromatographic experiments were performed on *Shimadzu LC-20* chromatographs with a refractometric detector. Chromatographic conditions: columns: 4.6 × 250 mm *Kromasil* 100-5C18; mobile phases 100% acetone; the rate of the mobile phase 0.8 mL/min, column temperature 30 °C, and retention time of squalene 8.25 min.

### 2.4. Characterization of the inclusion complexes

#### 2.4.1. Inclusion yield and squalene content

The content of squalene in the complex was determined by RP-HPLC using a calibration curve (0.05–10 mg/mL); LOQ: 0.12 mg/mL). Squalene extraction was obtained by mixing 20 mg of inclusion complex and 3 mL of acetone in a vial for 15 min. The mixture was centrifuged for acetone solution of squalene separation. The process was repeated three times to completely extract squalene in the complex, while the extraction completeness was controlled by comparing the IR spectra of βCD and the powder after extraction. The combined acetone solutions were used for squalene determination by RP-HPLC.

An inclusion yield and squalene content in the obtained complexes were calculated using the formula:


$$\begin{array}{l} Yield\%=\frac{complex,mg}{\left[(\text{squalene},\text{mg})+(\text{CD},\text{mg})\right]}100(\%)\\ \text{and squalene content},\;\%=\frac{released\;squalene,mg}{complex,mg}100(\%) \end{array}$$

#### 2.4.2. Fourier-transform infrared spectra analysis

FT-IR spectra were measured using a spectrometer *Shimadzu IR Prestige* with KBr pellets. The scans were acquired at a resolution of 4.0 cm^−1^ (from 450 to 4000 cm^−1^).

#### 2.4.3. X-ray diffractometry

The diffraction patterns of βCD, Me-βCD, and inclusion complexes were measured using an X-ray diffractometer Rigaku Ultima IV using detector DTEX/ULTRAT. CuKα radiation (λ = 1.5406 Å) was applied to measure the diffraction angles in the region 5° to 50° under a voltage of 40 kV and current of 30 mA and at a speed of 5°/min.

#### 2.4.4. Differential scanning calorimetry and thermal gravimetric analysis

Differential scanning calorimetry (DSC) measured the heat flow thermograms and thermal gravimetric analysis (TGA) of βCD, Me-βCD, and their inclusion complexes were performed using an SDT Q600 under argon atmosphere. The analysis was carried out from 25 to 500 °C at a heating rate of 10 °C min^−1^.

#### 2.4.5. Nuclear magnetic resonance spectroscopy analysis

^1^H NMR spectrums were measured on a JEOL JNM-ECA 600 at a frequency of 600 MHz at room temperature. βCD, Me-βCD, and inclusion complexes were dissolved in D_2_O, and squalene was dissolved in CDCl_3_. Chemical shifts were presented in ppm by using tetramethylsilane as an internal standard.

#### 2.4.6. Molecular modeling studies

The initial structure of molecules (squalene, βCD, Me-βCD, and IC) was built using Gaussian view 09. The starting geometries were performed by Gaussian 09 and were optimized by method MP3. The complexation energy ΔE_IC_ is calculated from the minimum energy structures using the Gaussian 09 molecular modeling package with method MM2, PM3, and DFT by the equation in the absence of solvent:


$$\Delta {\text{E}}_{\text{IC}}={\text{E}}_{\text{IC}}-{\text{E}}_{\text{host}}-{\text{E}}_{\text{gest.}}$$

E_IC_, E _host_, and E _gest_ represent the total energy of the inclusion complex, the free host (βCD or Me-βCD), and squalene molecules, respectively.

### 2.5. Experimental design and statistical analysis

The Face Centered Central Composite Design was applied to optimize the operating conditions. The molar ratio of β-cyclodextrin to squalene (X), an initial concentration of cyclodextrin (Y) (mM), and inclusion time (Z) (min) were selected as the independent factors, and the inclusion yield (A) and squalene content (B) in the complex were chosen as responses. The range of values of factors was chosen based on obtained results using the one-factor-at-a-time method. In this design, the randomized run order was created by Minitab software, including six replications at the central point.

The determination of the optimum set of operating conditions was archived using a quadratic model for each response and expressed according to the equation:


$$\text{Response}=\beta_{\text{o}}+\beta_{1}\text{X}+\beta_{2}\text{Y}+\beta_{3}\text{Z}+\beta_{11}{\text{X}}^{2}+\beta_{22}{\text{Y}}^{2}+\beta_{33}{\text{Z}}^{2}+\beta_{12}\text{XY}+\beta_{13}\text{XZ}+\beta_{23}\text{YZ}$$

Where β_o_ – intercept; β_i_ (i = 1, 2, 3) - the linear terms; β_ii_ (i = 1, 2, 3) - the quadratic terms; and β_ij_ - the interaction terms

For model analysis, significant factors that contributed to responses were eliminated at a 5% probability level, and the efficiency of the model was determined by evaluating the lack of fit and coefficient of determination (R^2^) that was generated by the software Minitab v.9.1.1 (Minitab Inc., State College, PA, USA).

## 3. Results and discussion

### 3.1. Optimization of inclusion complex production

The choice of optimal conditions for complex production depends on the nature of the inclusion complexes. The production was controlled by the inclusion yield and the squalene content in the complex and conducted at room temperature to prevent the destruction of polyene compounds. High-speed mechanical desperation was used for the substance “guests” of high hydrophobicity as squalene. The single-factor tests of the three factors were estimated, and the general levels of each factor were determined (Table 1). The Face Centered Central Composite Design was applied to optimize the condition in preparation. The molar ratio of β-cyclodextrin to squalene (X), an initial concentrate of cyclodextrin (Y) (mM), and inclusion time (Z) (min) were selected as independent factors, and the yield of complex (A) and squalene content (B) in the complex were chosen as responses. In this design, the randomized run order was created by Minitab software, including six replications at the central point. The range of βCD initial concentration was chosen based on the solubility in water of βCD.

The polynomial equation for yield complex and mass fraction of squalene in the complex are as follows:


$$\text{A}=-50.283+1.047\;\text{X}+14.347\;\text{Y}+5.368\;\text{Z}-0.1705\;{\text{X}}^{★}\text{X}-0.8737\;{\text{Y}}^{★}\text{Y}-0.4768\;{\text{Z}}^{★}\text{Z}+0.22500\;{\text{X}}^{★}\text{Y}-0.17031\;{\text{X}}^{★}\text{Z}+0.39062\;{\text{Y}}^{★}\text{Z}$$

Where R^2^ = 0.9998; R^2^(ajd)=0.9996


$$\text{B}=-12.079+0.8980\;\text{X}+2.8477\;\text{Y}+1.4693\;\text{Z}-0.08379\;{\text{X}}^{★}\text{X}-0.18004\;{\text{Y}}^{★}\text{Y}-0.10879\;{\text{Z}}^{★}\text{Z}+0.03148\;{\text{X}}^{★}\text{Y}-0.03883\;{\text{X}}^{★}\text{Z}+0.04242\;{\text{Y}}^{★}\text{Z}$$

Where R^2^ = 0.9999; R^2^(pred) = 0.9997

**Figure 2:**
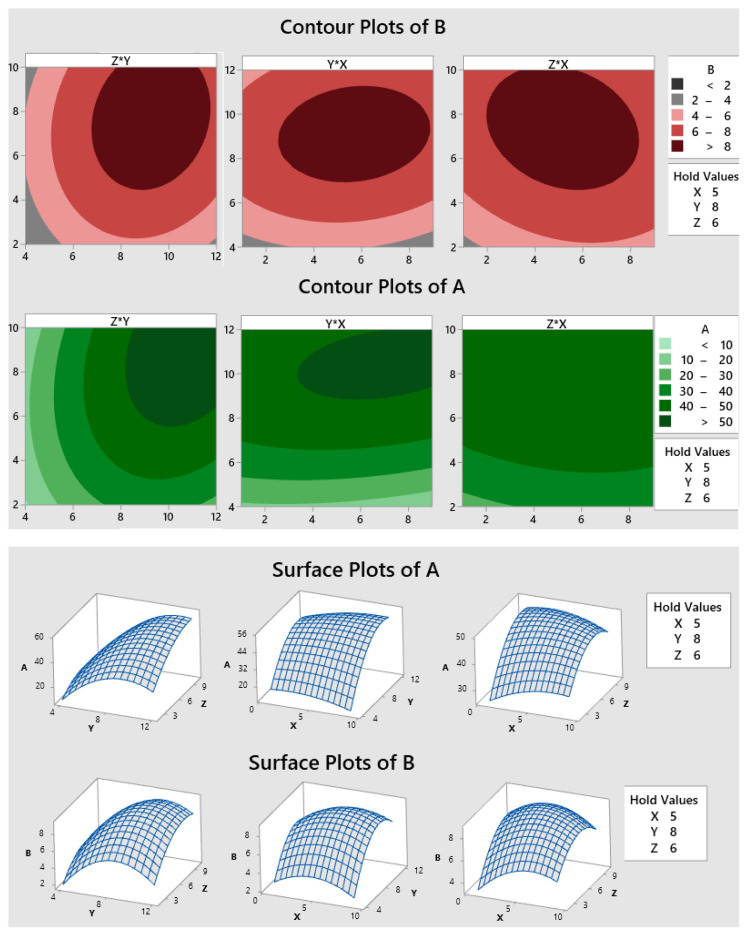
Contour graph and response surface plot illustrating the effect of three independent parameters on inclusion yield (A) and the mass fraction of squalene in complex (B).

Analysis of variance (ANOVA) of the quadratic regression models for yield complex and squalene content showed that both models were significant (p < 0.05). There was no significance in the “lack of fit” (p > 0.05) in each of the models indicating that the models could be used to predict the response. A complete analysis of variance for two responses was listed in the supplementary material (Tables S1 and S2). For both models, all linear coefficients of three parameters (X, Y, and Z) are positive, indicating a synergistic effect. The coefficient of the initial concentration of βCD (parameter Y) is the greatest in three independent parameters, showing the highest impact on the inclusion yield and squalene content compared to the parameters of inclusion time and the molar ratio. Similar results were obtained by Ren et al. [14] using Box–Behnken design to optimize the condition in preparation for the inclusion complex of Glaucocalyxin A sulfobutylether-β-cyclodextrin. In both cases, the process variables of initial concentrations (Y), molar ratio (X), inclusion time (Z), and all interaction terms were significant model terms (p values < 0.05). The combined effect of three independent parameters on two responses was shown in Figure 2.

As shown in Figure 2, maximization of inclusion yield was observed when the concentration of β-CD, the molar ratio of β-CD to squalene, and inclusion time varied between 9–12 mM, 3–9, and 6–10 min, respectively. A similar observation was also observed with the squalene content in the complex, describing optimum range values at a concentration of 7–11 mM β-CD, the initial molar ratio of β-CD to squalene 3–8, and inclusion time of 5–10 min. The inclusion yield and squalene content increased rapidly and gradually tended to equilibrium within 8 min. In addition, the yield and squalene content also increased quickly with an initial concentration of β-CD (in the range of 4–10 mM). The yield almost remains constant after 10 mM while the squalene content slightly decreases, related to the self-associating ability of molecules β-CD in the complex [15]. The molar ratio of β-CD to squalene on the yield and squalene content seems to be the same, rapidly declining in the range of 5–20 and unchanging in the range below 5.

**Figure 3:**
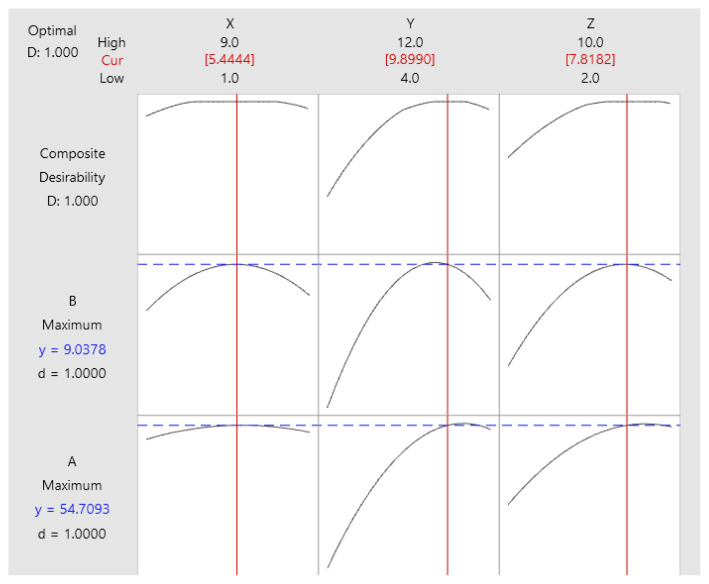
Plot showing the overall desirability of the model resulting in the highest inclusion yield and the mass fraction of squalene in the complex.

The simultaneous maximization of both responses was shown in Figure 3. By considering the actual test conditions, the initial concentration of βCD 9.9 mM, inclusion time of 7.8 min, and the molar ratio of βCD to squalene 5.4 were selected from the optimum experimental parameters. The preparation was controlled under these conditions; a mean inclusion yield of 54.3% ± 0.5% (n = 3) and squalene content of 9.01% ± 0.06% (n = 3) was achieved. The obtained results were slightly lower than the theoretical value within the error range. The squalene content in the inclusion complex corresponding to the molar ratio of βCD to squalene was approximately 3:1. This conclusion is entirely consistent with the study results from molar ratio of βCD with squalene in the inclusion complex, prepared by coprecipitation method [16].

The significant difference in the complexion of squalene with Me-β-CD is that no precipitate forms in the initial mixture, i.e. such complexes are more efficiently dispersed in water. Therefore, single-factor tests of the molar ratio of Me-β-CD to squalene were estimated with Me-βCD concentration of 10 mM. The archived results showed that squalene content in the complex increases to 30.5% with the increased volume of squalene. Then, the content of squalene is practically maintained. The squalene content in the squalene-Me-β-CD complex is significantly higher than in the squalene-β-CD complex. In addition, for the squalene-Me-β-CD complex received by freezing-dried mixed solution, the content of squalene was total content squalene not only included in the cyclodextrin cavity but also on the surface of the cavity, which was confirmed by the results of thermal analysis.

### 3.2. FTIR spectra analysis

**Figure 4:**
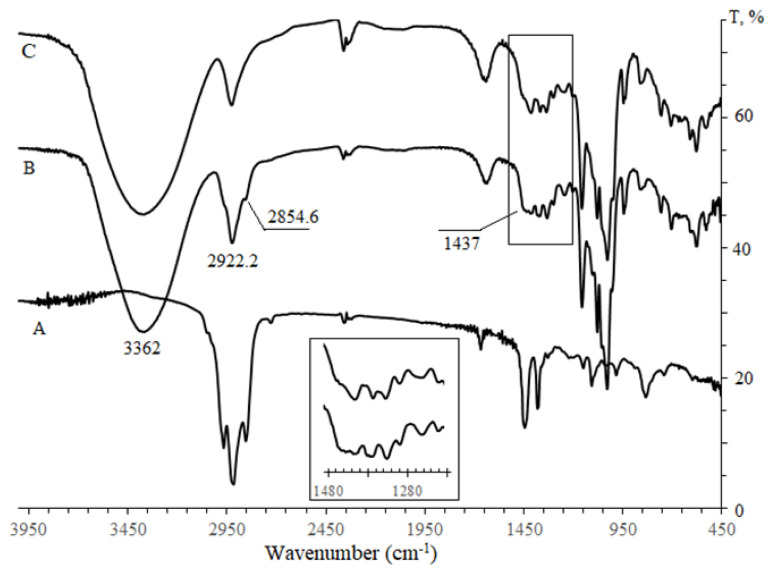
FTIR spectra of squalene- βCD inclusion complex (B), squalene (A), and βCD (C).

FTIR spectra of pure squalene, βCD, Me-βCD, and physical mixtures of both cyclodextrins with molecular gest, prepared with a similar ratio in complexes, were compared with two obtained complex spectrums. Pure squalene and physical mixtures exhibited characteristic peaks at 2902.8, 2972.3 and 2991.5 cm^−1^ corresponding to stretching absorption of C-H, and at 1496 and 1425 cm^−1^ relating to bending vibrations of C-H bonds (as shown in Figure 2S). However, as shown in Figure 4, in the infrared spectrum of the complex, all characteristic βCD bands were preserved, and squalene bands were added. During the formation of the complex, an increase in the absorption band intensity was observed in the region of 2800–3000 cm^−1^, which is characteristic of Csp^3^-H (stretching vibrations) of CH, CH_2,_ and CH_3_ groups (added from squalene band). Differences in the IR spectra are typical due to changes or loss of vibrating and bending of the guest molecule during complex formation. For the inclusion complex, the group of cyclic ether could be observed for stretching vibration of C–O–C in the region of 1025–1158 cm^−1^ (1028, 1076, 1153 cm^−1^); in the region of 1480–1190 cm^−1^ (1452, 1413, 1372, 1332, 1299, 1244 cm^−1^) relating to the deformation vibrations of the C–H bonds the complex band also differs markedly from that of β-CD. The band at 3404 cm^−1^ for O-H groups experienced a significant broadening of the spectra for physical mixture and pure cyclodextrins, and peaks were shifted toward the lower frequency of 3350 cm^−1^ in the complex. The change indicated the decrease of hydrogen bonds in complex formation.

The same change was found in the IR spectra of the squalene–methyl-βCD complex compared to that of squalene and Me-βCD (see Figure 1S in supplementary). The changes were related to the superposition of corresponding bands in initial substances.

### 3.3. X-ray diffraction and thermal analysis

The formation of an inclusion complex, instead of a physical mixture of β-CD and squalene, was confirmed by analysis of X-ray diffraction patterns and thermal stability properties. The samples of inclusion complexes and cyclodextrins prepared from the solution after drying were analyzed.

**Figure 5:**
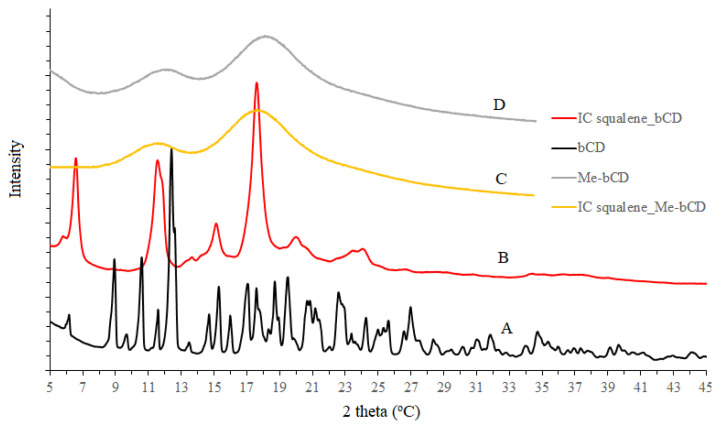
Diffraction patterns of βCD (A), squalene-βCD complex (B), squalene-Me-βCD complex (C), and Me-βCD (D).

It was found that the formation of the complex changes the initial crystal lattice of β-CD and, therefore, the X-ray diffraction pattern. The βCD spectrum indicates high crystallinity, due to characteristic sharp peaks at (041), (141), (180), (042), (162), (222), (223), and (044) (pattern A, Figure 5), according to XRD results [17]. The diffraction spectra of βCD were dissimilar to the spectrum of the inclusion complex, indicating a more formless structure with the peaks appearing at 6.62°, 11.62°, 15.16°, and 17.58° (as shown in pattern B, Figure 5). Since the spectra of the complex showed new crystal peaks, it was possible to predict the phase transition for the complex during the preparation process. Conversion of the new crystal type for βCD revealed the formation of an inclusion complex between squalene and β-CD. The intermolecular interaction of squalene and βCD may disrupt the initial organization of βCD molecules and transform crystal lattices.

The diffraction pattern of Me-βCD (pattern D, Figure 5) has two broad peaks at 12.16° and 18.24°, indicating an amorphous structure. Meanwhile, the spectrum of complex squalene-Me-βCD slightly differed with peaks at 11.74° and 17.74°, showing the formation of inclusion complex between squalene with Me-βCD.

**Figure 6:**
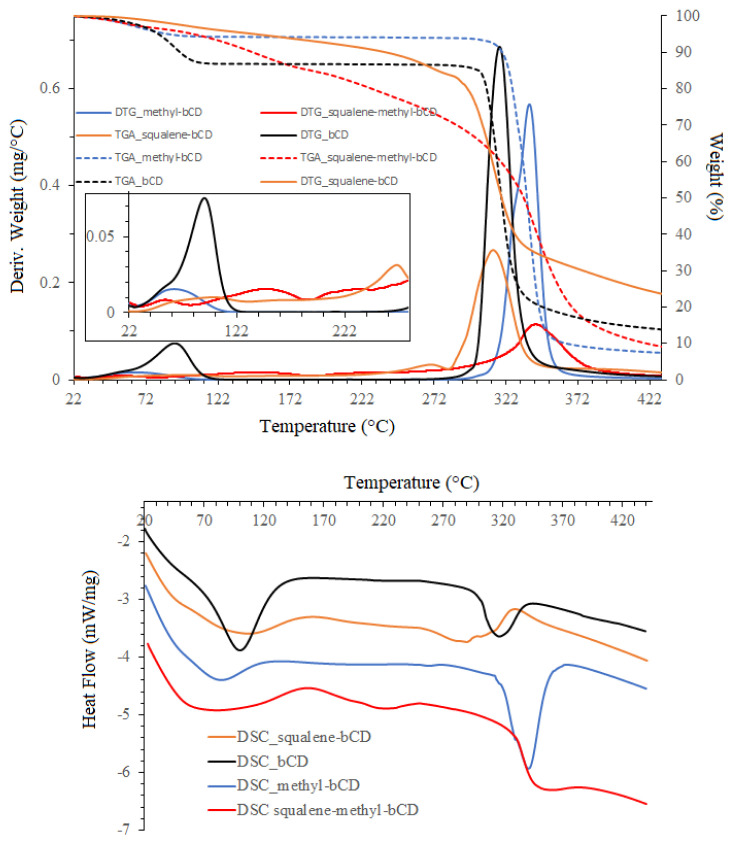
Thermogravimetric analysis (A) and differential scanning calorimetry (B) of βCD, Me-βCD, and their complexes.

On the DSC thermogram of βCD (Figure 6A), there is a big endothermic peak in the range of 75–136 °C, associated with the process of dehydration of cyclodextrin and evaporation of water molecules, corresponding to results in weight loss during thermogravimetric analysis at 95.7 °C. Up to 280 °C, no further weight loss was found, then the decomposition of β-CD started over 282 °C. Meanwhile, for the squalene–beta-cyclodextrin sample, an endothermic peak in the range of 254–290 °C, characteristic according to the observed results for the DSC and TGA analysis (Figure 6B) of βCD inclusion complexes with essential oil [17], was replaced by the exothermic peak resulting by complicated processes of complex decomposition indicating the interaction between the guest molecule and βCD in starting material. In this case, at first, squalene may be evaporated (exothermic effect) accomplished with released beta-cyclodextrin recrystallization (exothermic process) and subsequent squalene evaporation and beta-cyclodextrin destruction.

Similar results of DSC and TGA were also obtained in the case of complexes squalene-Me-βCD. The TGA/DTG curves of Me-βCD appeared to have a broad peak at 66.8 °C, relating to dehydration. A similar peak was observed on DTG curves of inclusion complex with a slightly lower temperature at 56.9 °C. The comparison of DTG curves of Me-βCD and its complex showed that excepting peaks at 314–370 °C corresponding to the decomposition of Me-βCD, on DSC curves of the complex appeared two new peaks, indicating the decomposition of the squalene from the Me-βCD cavity (from 193–251 °C) and evaporation decomposition of the squalene, which is on the surface of the cavity (from 112–187 °C). The absence of Me-βCD recrystallization makes this band only endothermic in contrast to the squalene–beta-cyclodextrin sample.

### 3.4. ^1^H NMR spectrum analysis

**Figure 7:**
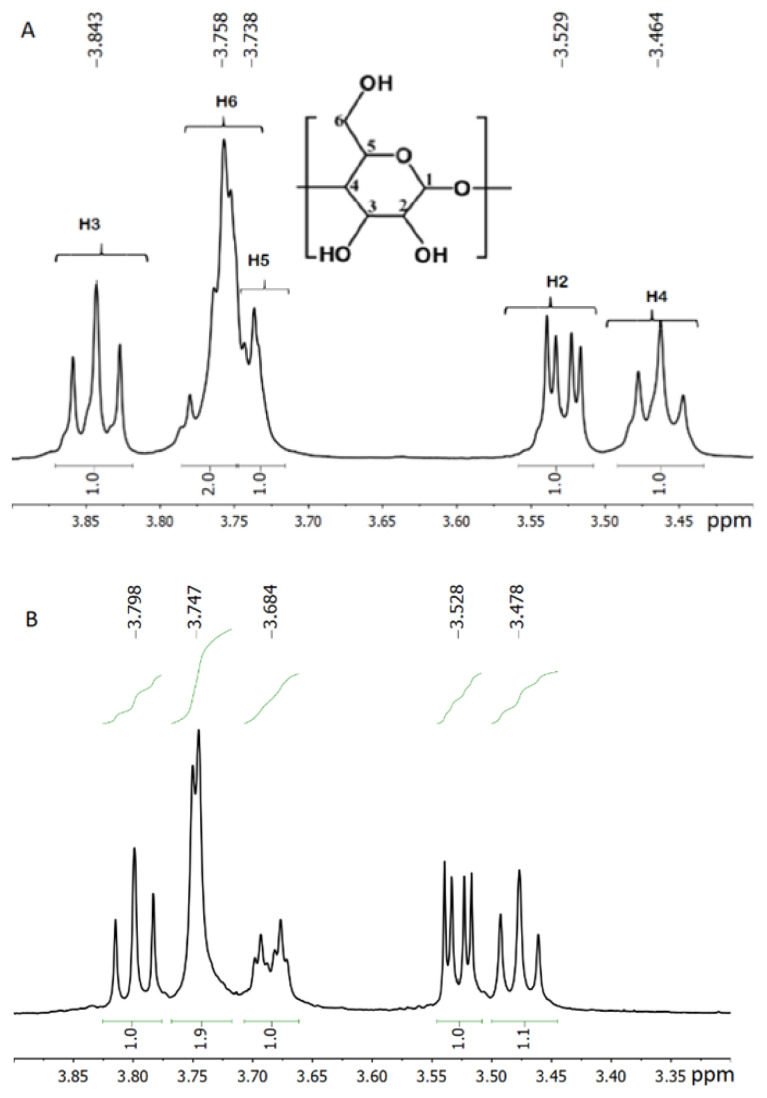
600 MHz ^1^H-NMR spectra of cyclodextrin(A) and inclusion complex of squalene with βCD (B) in D_2_O ranging from 3.35 to 4.0 ppm.

^1^H NMR spectra of squalene, Me-βCD, βCD, and two inclusion complexes were analyzed. Proton signals of CH_3_, CH_2,_ and =CH groups of squalene appeared at 1.6–1.75, 1.95–2.15, and 5.1–5.2 ppm, respectively, whereas almost proton signals of both cyclodextrins located at 3.0–5.8 ppm. Compared to pure squalene, no significant chemical shifts of the protons of two inclusion complexes were found, indicating that most of the proton signals of squalene and cyclodextrins were unaffected by the complexation. In ^1^H NMR spectra of inclusion complexes, the presence of squalene in two inclusion complexes was observed in the range of 1.2–2.5 ppm, related to the protons in CH_3_ and CH_2_ groups. Low-intensive peaks with chemical shifts at 5.1–6.2 ppm correspond to protons bonded to C=C. However, ^1^H NMR spectroscopy was a valuable method for characterizing inclusion complexes due to the significant change of chemical shifts of H-3 and H-5 protons in the spectra of complexes oriented inside the hydrophobic cyclodextrin cavity [19]. Seven D-glucose units with similar conformations and hexagonal symmetry were recognized in ^1^H-NMR spectrum (as shown in Figure 7, A). The presence of squalene in the inclusion complex causing the changes of chemical shift of the spectrum squalene-βCD complex was observed (Figure 7, B). The changes in the chemical shift (Δδ) of protons on β-CD structures caused by the inclusion behavior are shown in Table 2. The greatest change in chemical shifts between the spectra of β-CD and the complex was found for H-1 and H-5 protons inside the cavity. Otherwise, a slight change in the outside protons (H1, H2) was observed. This means that the interaction between the host and guest molecule occurs when the guest molecule, according to literature data [19] is located in the nonpolar cavity of cyclodextrin.

**Figure 8:**
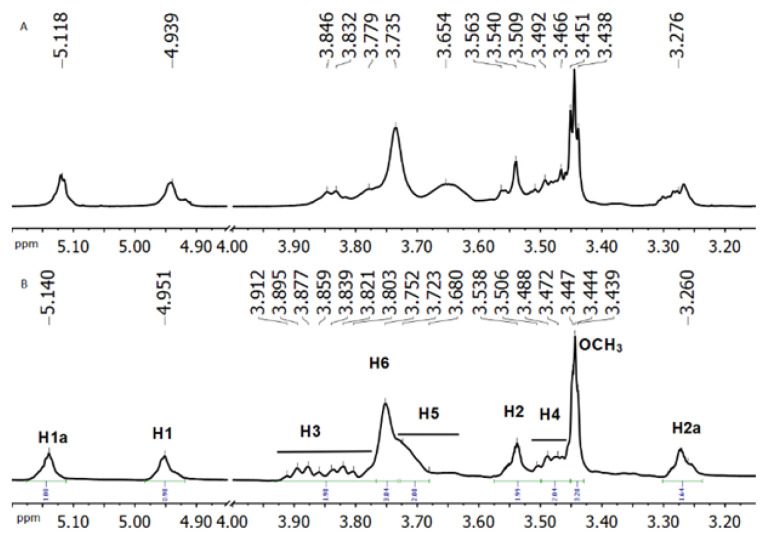
1H-NMR spectra of Me-βCD (B) and squalene-Me-βCD complex (A) in D_2_O.

In the ^1^H-NMR spectrum of Me-βCD (Figure 8), two peaks with the highest absolute shift value were found, indicating a mixture of molecules of randomly methylated beta-cyclodextrin with an irregular position of methylation; the same is true for two types of H-2 protons. The peak of the methoxy group (OCH_3_) at 3.444 ppm can be assigned to proton H-2 [20]. The results showed that the used Me-βCD is (2-O-methyl)-β-cyclodextrin with an average degree of substitution of 0.52 (as shown in Figure 1), according to the result [21]. The cyclodextrin protons’ chemical shifts, δ, as well as the differences for starting cyclodextrin and for obtained complexes, Δδ, for squalene–Me-βCD are shown in Table 2. The most significant upfield shifts of the H-3, H-5 protons with similar magnitude were observed, indicating the squalene molecule is involved in the Me-βCD cavity.

### 3.5. Molecular modeling

**Figure 9:**
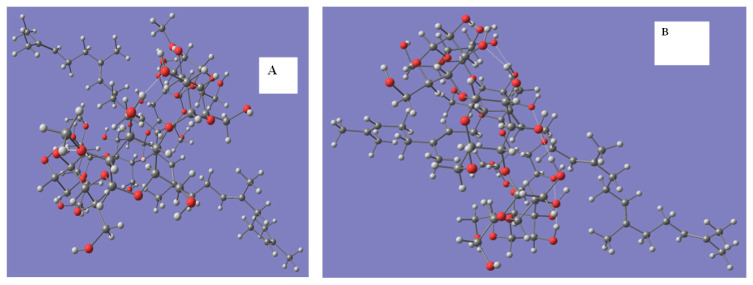
Geometric structures of the supramolecular complex of squalene–βCD (B) and squalene–Me-βCD using DFT method.

The inclusion complexes of squalene with βCD and Me-βCD were studied by computational MM2, PM3, and DFT methods. The molecular graphics of both inclusion complexes calculated by the DFT method are shown in Figure 9.

As a result, squalene molecular successfully inserted into the cavity of cyclodextrin, and the maximization of the ratio of β-CD and squalene was 3:1 (as shown in Figure S2 in supplementary), which corresponds to experimentally obtained results.

Calculating by three methods (as shown in Table 3), the energy of the complexes decreases when the squalene molecule is involved in the cyclodextrin cavity, indicating that weak intermolecular interactions contribute to stabilizing complexes.

## 4. Conclusion

The supramolecular complex formation in an aqueous solution between squalene with methyl-βCD and βCD was supported by FT-IR, XRD, DSC, TGA, and ^1^H-NMR analysis. According to these results, the complexes are formed by the inclusion of the squalene into the cyclodextrin cavity, confirmed by molecular modeling.

Using Response Surface Methodology, the optimal conditions for forming complexes of β-CD and Me-β-CD with squalene were studied. For complex squalene-β-CD, the optimal conditions of initial concentration of βCD, inclusion time, and the mole ratio of βCD to squalene were 9.9 mM, 7.8 min, and 5.4. Under these conditions, a mean yield of the complex of 54.5% and squalene content of 9.01% was achieved. The squalene content in the inclusion complex corresponding to the molar ratio of βCD to squalene was approximate 3:1. For Squalene-Me-β-CD complex, complexes are more efficiently dispersed in water, and squalene content in the complex increases to 30.5%, which may be used in pharmacy and cosmetics.

## Electronic supporting information

**Table S1:** Analysis of variance for the response surface quadratic model of the yield of complex (A).

Source	DF	Adj SS	Adj MS	F-Value	P-Value
Model	10	5736.71	573.67	4287.15	0.000
Blocks	1	0.08	0.08	0.57	0.469
Linear	3	2948.35	982.78	7344.54	0.000
X	1	2.30	2.30	17.22	0.002
Y	1	2356.22	2356.22	17,608.53	0.000
Z	1	589.82	589.82	4407.87	0.000
Square	3	1805.18	601.73	4496.83	0.000
X*X	1	19.98	19.98	149.33	0.000
Y*Y	1	524.49	524.49	3919.58	0.000
Z*Z	1	156.20	156.20	1167.33	0.000
2-Way interaction	3	475.58	158.53	1184.71	0.000
X*Y	1	103.68	103.68	774.82	0.000
X*Z	1	59.41	59.41	443.95	0.000
Y*Z	1	312.50	312.50	2335.37	0.000
Error	9	1.20	0.13		
Lack-of-fit	5	0.99	0.20	3.73	0.113
Pure error	4	0.21	0.05		
Total	19	5737.91			

**Table S2:** Analysis of variance for the response surface quadratic model of squalene content (B).

Source	DF	Adj SS	Adj MS	F-Value	P-Value
Model	10	180.093	18.0093	7195.82	0.000
Blocks	1	0.005	0.0049	1.95	0.196
Linear	3	39.258	13.0860	5228.68	0.000
X	1	0.999	0.9986	398.99	0.000
Y	1	22.983	22.9826	9182.96	0.000
Z	1	15.277	15.2770	6104.09	0.000
Square	3	103.268	34.4227	13,754.02	0.000
X*X	1	4.825	4.8247	1927.77	0.000
Y*Y	1	22.275	22.2745	8900.06	0.000
Z*Z	1	8.133	8.1331	3249.69	0.000
2-Way interaction	3	8.803	2.9344	1172.49	0.000
X*Y	1	2.030	2.0301	811.16	0.000
X*Z	1	3.088	3.0876	1233.69	0.000
Y*Z	1	3.686	3.6856	1472.63	0.000
Error	9	0.023	0.0025		
Lack-of-fit	5	0.020	0.0040	5.94	0.055
Pure error	4	0.003	0.0007		
Total	19	180.115			

Figure 1S IR spectra of squalene-Me-βCD inclusion complex (A), squalene (C), and Me-βCD (B).

Figure 2S IR spectra of physical mixtures of squalene with Me-βCD (35:75, w/w) (1) and squalene with βCD (14:86, w/w) (2).

Figure 3S Geometric structures of the supramolecular complex of squalene–βCD (ratio 1:3) (B) using DFT method.

Figure 4S Yield complex with different inclusion time of squalene-β-CD inclusion complex (20 μL squalene and 20 mL solution of βCD 0.01M in water).

Figure 5S Inclusion yield (a) and squalene content (b) in squalene-β-CD inclusion complex with different volume of added squalene (in 20 mL solution of βCD 10 mM).

Figure 6S Squalene content of squalene-Me-β-CD inclusion complex with different volumes of added squalene (in 20 mL solution of βCD 10 mM).

Figure 7S ^1^H NMR spectrum of squalene in CDCl_3_.

Figure 8S ^1^H NMR spectrum of squalene_Me-βCD complex in D_2_O.

Figure 9S ^1^H NMR spectrum of squalene_ βCD complex in D_2_O.

Figure 10S Thermogravimetric analysis of pure squalene.

## Figures and Tables

**Table 1:** Experimental design for response surface analysis.

Run order	Block	Independent factors*			Response**	
		X	Y(mM)	Z (min)	A (%)	B (%)
16	2	5.0	12.0	6.0	48.9	7.30
19	2	5.0	8.0	6.0	47.2	8.55
15	2	5.0	4.0	6.0	17.8	4.13
18	2	5.0	8.0	10.0	47.8	8.10
13	2	1.0	8.0	6.0	44.5	6.91
14	2	9.0	8.0	6.0	44.7	7.60
17	2	5.0	8.0	2.0	31.6	5.61
20	2	5.0	8.0	6.0	47.3	8.57
12	1	5.0	8.0	6.0	47.4	8.63
8	1	9.0	12.0	10.0	53.3	6.24
9	1	5.0	8.0	6.0	46.8	8.66
3	1	1.0	12.0	2.0	17.3	0.79
6	1	9.0	4.0	10.0	3.2	0.92
4	1	9.0	12.0	2.0	31.3	3.70
7	1	1.0	12.0	10.0	50.6	5.90
2	1	9.0	4.0	2.0	5.8	1.01
10	1	5.0	8.0	6.0	47.2	8.59
11	1	5.0	8.0	6.0	47.3	8.63
1	1	1.0	4.0	2.0	6.6	0.20
5	1	1.0	4.0	10.0	14.5	2.51

* X, Y, and Z represented the molar ratio of β-cyclodextrin to squalene, initial concentrate of cyclodextrin (mM), and inclusion time (Z) (min), respectively

** A and B represented the yield of complex and squalene content

**Table 2:** Chemical shifts δ (ppm) βCD, Me- βCD, and their complex with squalene in ^1^H NMR spectrum.

δ (βCD)	Δ (Me-βCD)	δ (complex) squalene-Me-βCD	δ (complex) squalene- βCD	Δδ_1_	Δδ_2_	Proton
-	5.141	5.118	-	-	−0.023	H-1a
4.949	4.951	4.939	4.944	−0.005	−0.012	H-1
***3.843***	***3.865***	***3.831***	***3.798***	−***0.045***	−***0.034***	***H-3***
3.758	3.752	3.735	3.747	−0.011	−0.017	H6, H6′
***3.738***	***3.723***	***3.654***	***3.684***	−***0.054***	−***0.069***	***H-5***
3.529	3.538	3.548	3.528	−0.001	0.010	H-2
3.464	3.488	3.485	3.478	0.014	−0.003	H-4
-	3.260	3.276	-	-	0.016	H-2a
-	3.444	3.445	-	-	0.001	OCH_3_(2)

Δδ_1_ = δ_complex_ (squalene-βCD)- δ (βCD) and Δδ_2_ = δ_complex_ (squalene-Me-βCD)- δ (Me-βCD)

**Table 3:** AM1, PM3, and DFT calculated parameters of βCD, Me- βCD, and their complexes with squalene.

Method	PM3	AM1	DFT
E(Me-βCD) (Kcal/mol)	−1428.43	−1628.46	−2,780,530.03
E(βCD) (Kcal/mol)	−1458.76	−1657.77	−2,681,910.56
E(Squalene) (Kcal/mol)	−29.41	−34.41	−736,044.25
E_IC_(squalene-βCD) (Kcal/mol)	−1514.59	−1667.61	−3,417,963.17
E_IC_(squalene-Me-βCD) (Kcal/mol)	−1484.32	−1695.85	−3,516,578.78
ΔE_IC_(squalene-βCD) (Kcal/mol)	−26.42	−3.68	−8.36
ΔE_IC_(squalene-Me-βCD) (Kcal/mol)	−26.48	−4.75	−4.50

## References

[1] Passi S de Pità O Puddu P Littarru GP Lipophilic antioxidants in human sebum and aging Free Radical Research 2002 36 4 471 478 10.1080/10715760290021342 12069113

[2] Lozano-Grande MA Gorinstein S Espitia-Rangel E Dávila-Ortiz G Martínez-Ayala AL Plant sources, extraction methods, and uses of squalene International Journal of Agronomy 2018 13 10.1155/2018/1829160

[3] Reddy LH Couvreur P A natural triterpene for use in disease management and therapy Advanced Drug Delivery Reviews 2009 61 15 1412 1426 10.1016/j.addr.2009.09.005 19804806

[4] Huang ZR Lin YK Fang JY Biological and Pharmacological Activities of Squalene and Related Compounds: Potential Uses in Cosmetic Dermatology Molecules 2009 14 1 540 554 10.3390/molecules14010540 19169201 PMC6253993

[5] Mudiyanselage SE Elsner P Thiele JJ Hamburger M Ultraviolet A Induces generation of squalene monohydroperoxide isomers in human sebum and skin surface lipids In Vitro and In Vivo Journal of Investigative Dermatology 2003 120 6 915 922 10.1046/j.1523-1747.2003.12233.x 12787115

[6] Farvin KH Anandan R Kumar SH Shiny KS Mathew S Cardioprotective effect of squalene on lipid profile in isoprenaline-induced myocardial infarction in rats Journal of Medicinal Food 2006 9 4 531 536 10.1089/jmf.2006.9.531 17201641

[7] Das B Yeger H Baruchel H Freedman MH Koren G In vitro cytoprotective activity of squalene on a bone marrow versus neuroblastoma model of cisplatin-induced toxicity implications in cancer chemotherapy European Journal of Cancer 2003 39 17 2556 2565 10.1016/j.ejca.2003.07.002 14602142

[8] Newmark HL Squalene, olive oil, and cancer risk. Review and hypothesis Annals of the New York Academy of Sciences 1999 889 193 203 10668494 10.1111/j.1749-6632.1999.tb08735.x

[9] Del Valle EMM Cyclodextrins and their uses: a review Process Biochemistry 2004 39 9 1033 1046 10.1016/S0032-9592(03)00258-9

[10] Tiwari G Tiwari R Rai AK Cyclodextrins in delivery systems: Applications Journal of Pharmacy and Bioallied Sciences 2010 2 2 72 79 10.4103/0975-7406.67003 21814436 PMC3147107

[11] Tian B liu Y Cyclodextrin-active natural compounds in food applications: a review of antibacterial Turkish Journal of Chemistry 2021 45 6 1707 1724 10.3906/kim-2106-51 PMC10734723 38144603

[12] Higashi T Motoyama K Li J Cyclodextrin-based catenanes and polycatenanes Journal of Inclusion Phenomena and Macrocyclic Chemistry 2022 102 569 575 10.1007/s10847-022-01143-4

[13] Loftsson T Brewster ME Pharmaceutical Applications of Cyclodextrins. 1. Drug Solubilization and Stabilization Journal of Pharmaceutical Sciences 1996 85 10 1017 1025 10.1021/js950534b 8897265

[14] Ren L Wang J Chen G Preparation, optimization of the inclusion complex of glaucocalyxin A with sulfobutylether-β-cyclodextrin and antitumor study Drug Delivery 2019 26 1 309 317 10.1080/10717544.2019.1568623 30896265 PMC6442205

[15] Loftsson T Magnúsdóttir A Másson M Sigurjónsdóttir JF Self-Association and Cyclodextrin Solubilization of Drugs Journal of Pharmaceutical Sciences 2002 91 11 2307 2316 10.1002/jps.10226 12379916

[16] Higashi T Tanaka H Yoshimatsu A Ikeda H Arima K Improvement of pharmaceutical properties of isoprenoid compounds through the formation of cyclodextrin pseudorotaxane-like supramolecules Chemical and Pharmaceutical Bulletin 2016 64 4 340 345 10.1248/cpb.c15-00931 26852798

[17] Ren W Yu X Wang S Blasier R Markel DC Cyclodextrin-erythromycin complexes as a drug delivery device for orthopedic application International Journal of Nanomedicine 2011 6 3173 3186 22228990 10.2147/IJN.S23530 PMC3252670

[18] Fernandes LP Éhen Z Moura TF Novák C Sztatisz J Characterization of Lippia sidoides oil extract-b-cyclodextrin complexes using combined thermoanalytical techniques Journal of Thermal Analysis and Calorimetry 2004 78 2 557 573 10.1023/B:JTAN.0000

[19] Gomes LMM Petito N Costa VG Falcão DQ Lima Araújo de KG Inclusion complexes of red bell pepper pigments with β-cyclodextrin: Preparation, characterisation and application as natural colorant in yogurt Food Chemistry 2014 148 428 436 10.1016/j.foodchem.2012.09.065 24262579

[20] Johnson JR Shankland N Sadler IH Full assignment of the proton and carbon-13 nmr spectra of 2,3,6 -tri-o-methyl-β-cyclodextrin Tetrahedron 1985 41 15 3147 3152 10.1016/S0040-4020(01)96669-4

[21] Coisne C Dorothée HV Boucau MC Hachani J Sébastien T β-Cyclodextrins Decrease Cholesterol Release and ABC-Associated Transporter Expression in Smooth Muscle Cells and Aortic Endothelial Cells Frontiers in Physiology 2016 7 185 10.3389/fphys.2016.00185 27252658 PMC4879322

