# Supplementary figures and images for: Inclusion complexes of squalene with beta-cyclodextrin and methyl-beta-cyclodextrin: preparation and characterization
Source: Turk J Chem. 2022 Dec 29;47(1):294–306. doi: 10.55730/1300-0527.3537 (PMC10504019; doi:10.55730/1300-0527.3537)

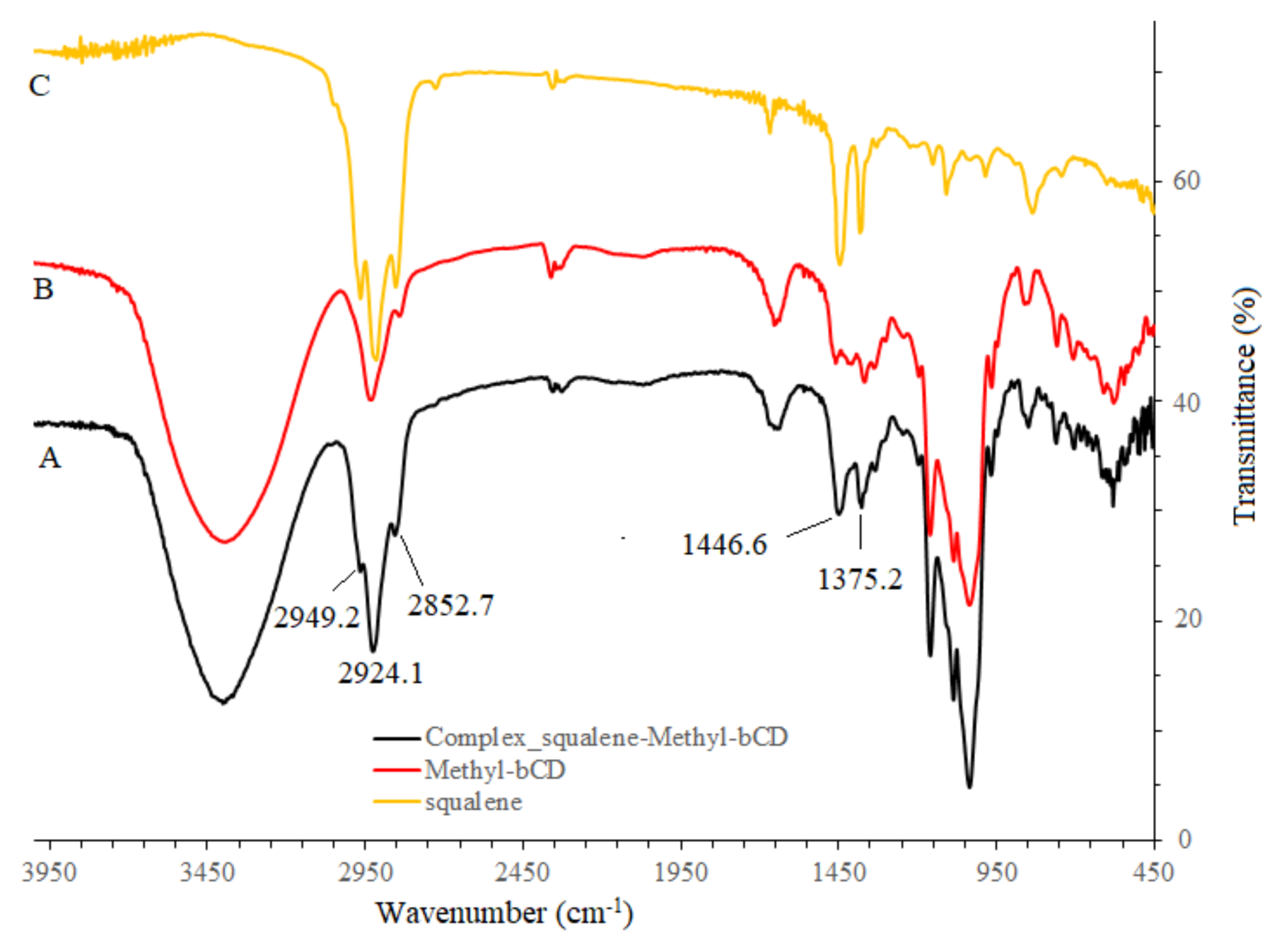

Supplement: Figure 1S — IR spectra of squalene-Me-βCD inclusion complex (A), squalene (C), and Me-βCD (B). [file turkjchem-47-1-294s1.tif]

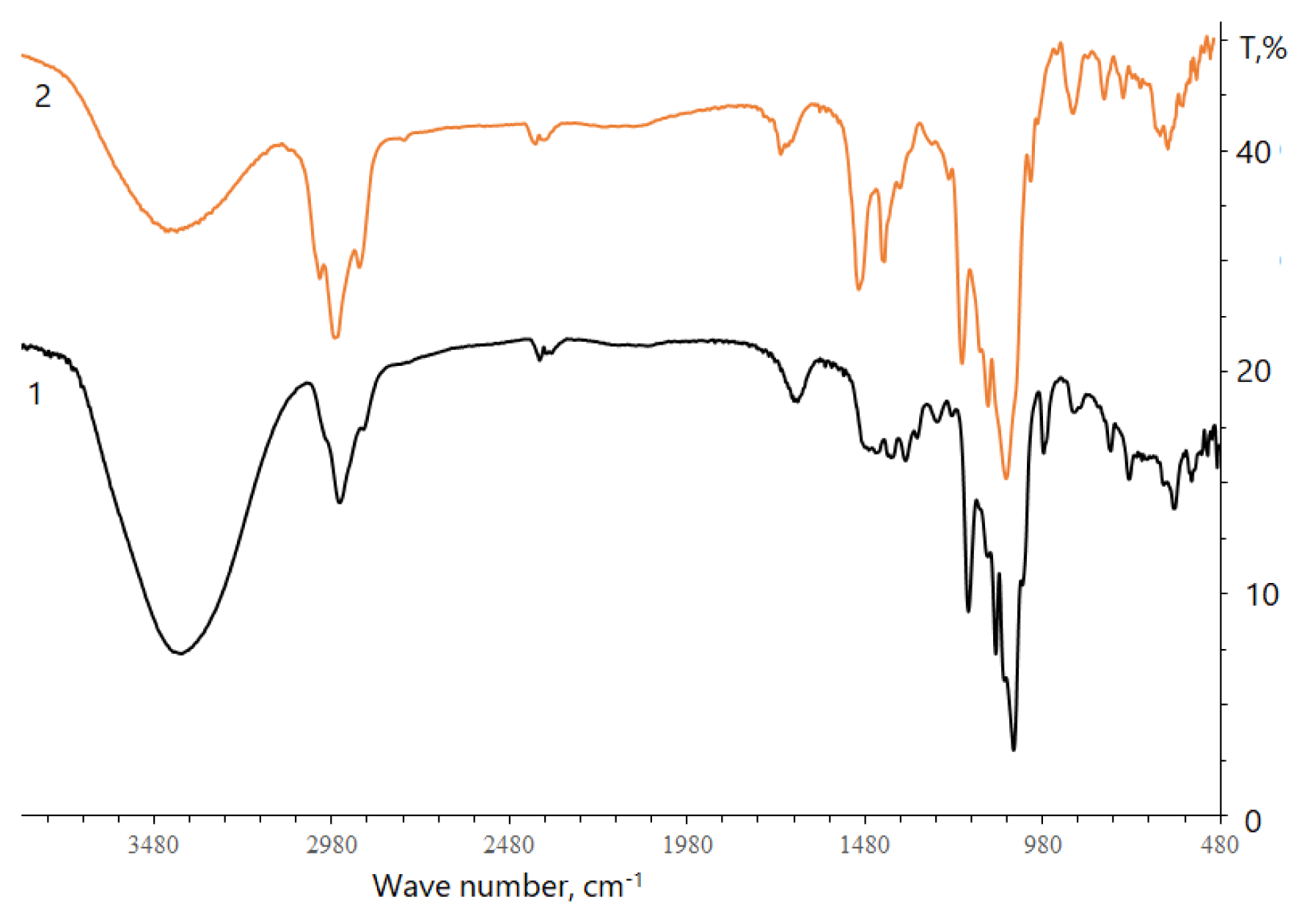

Supplement: Figure 2S — IR spectra of physical mixtures of squalene with Me-βCD (35:75, w/w) (1) and squalene with βCD (14:86, w/w) (2). [file turkjchem-47-1-294s2.tif]

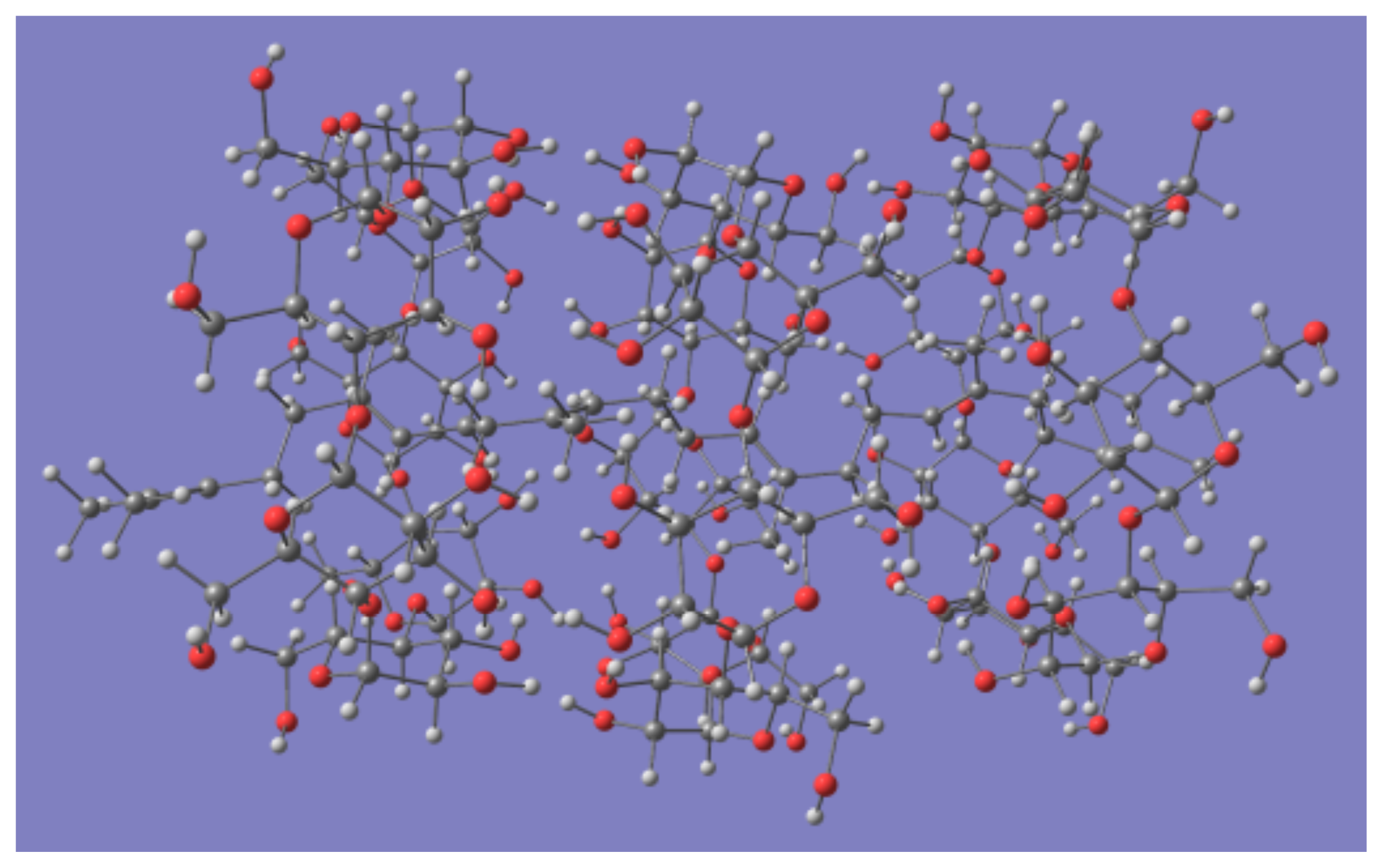

Supplement: Figure 3S — Geometric structures of the supramolecular complex of squalene–βCD (ratio 1:3) (B) using DFT method. [file turkjchem-47-1-294s3.tif]

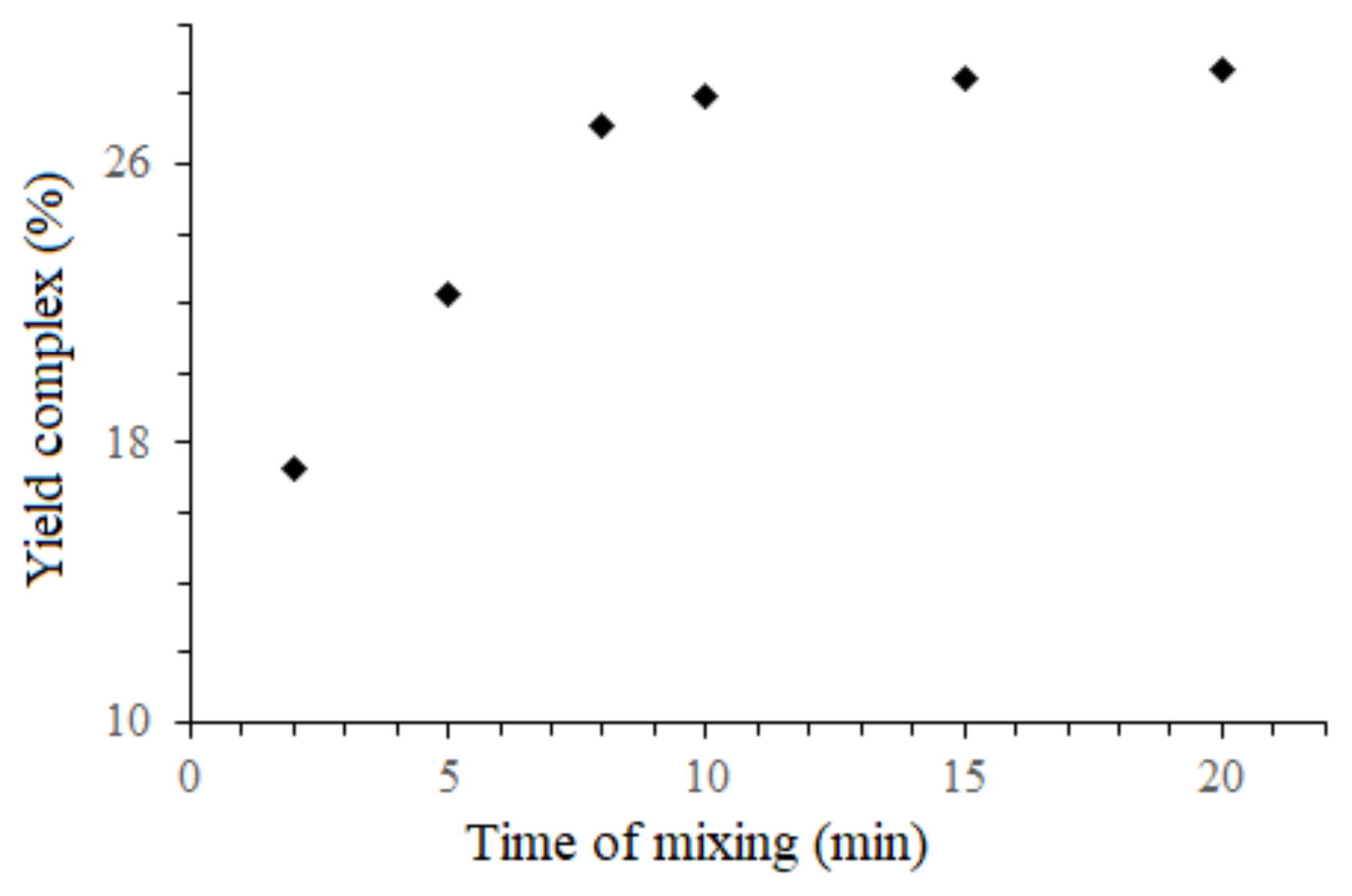

Supplement: Figure 4S — Yield complex with different inclusion time of squalene-β-CD inclusion complex (20 μL squalene and 20 mL solution of βCD 0.01M in water). [file turkjchem-47-1-294s4.tif]

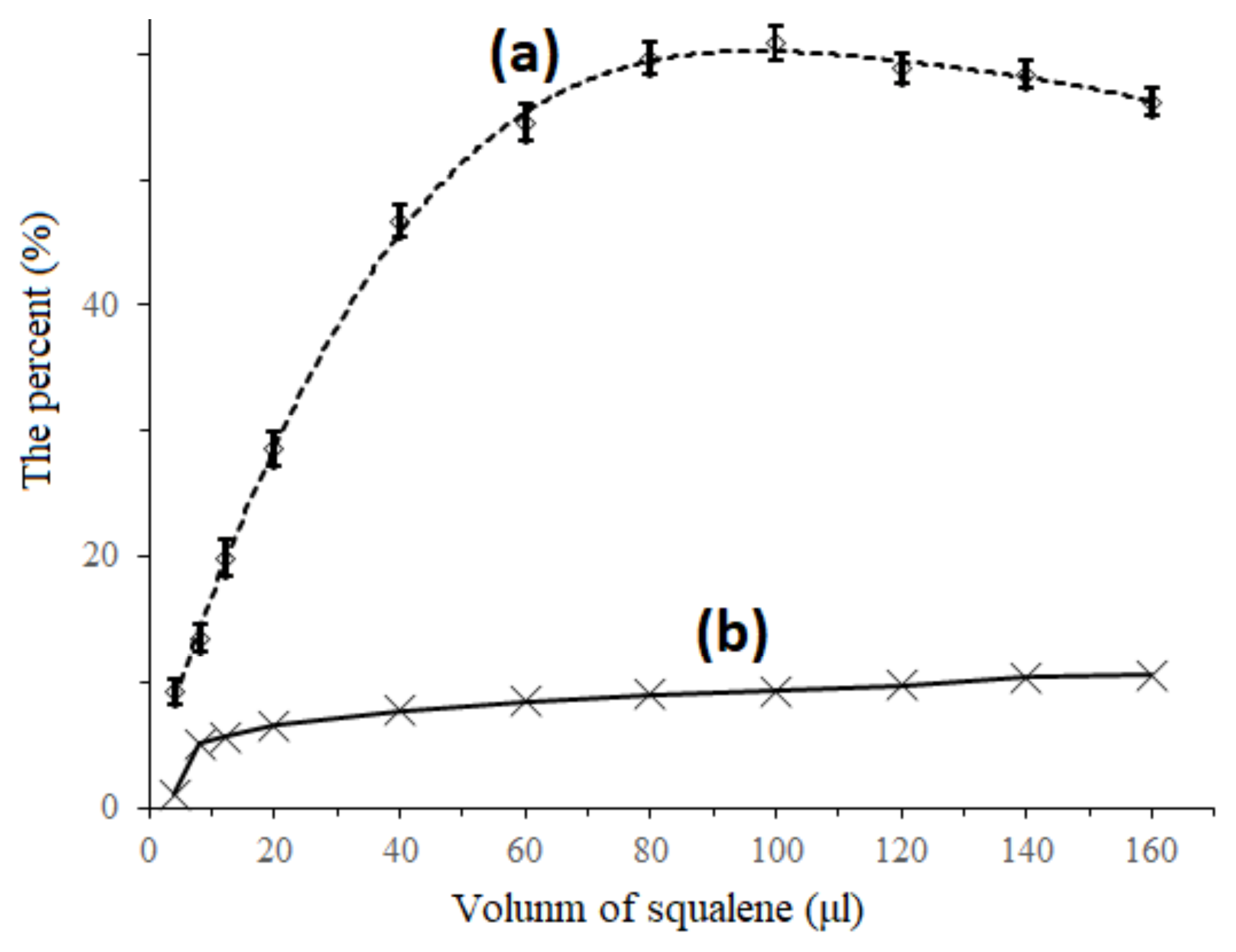

Supplement: Figure 5S — Inclusion yield (a) and squalene content (b) in squalene-β-CD inclusion complex with different volume of added squalene (in 20 mL solution of βCD 10 mM). [file turkjchem-47-1-294s5.tif]

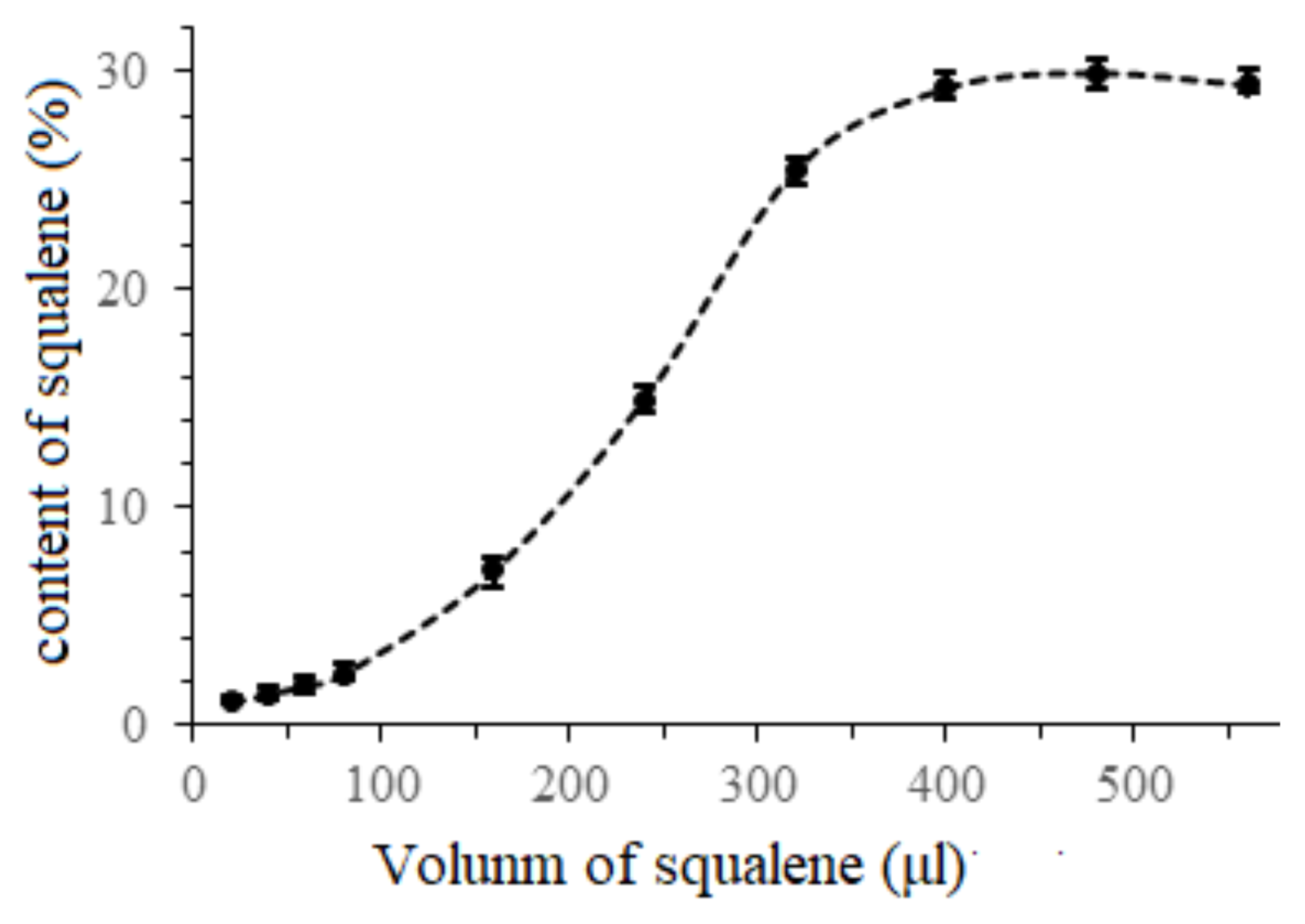

Supplement: Figure 6S — Squalene content of squalene-Me-β-CD inclusion complex with different volumes of added squalene (in 20 mL solution of βCD 10 mM). [file turkjchem-47-1-294s6.tif]

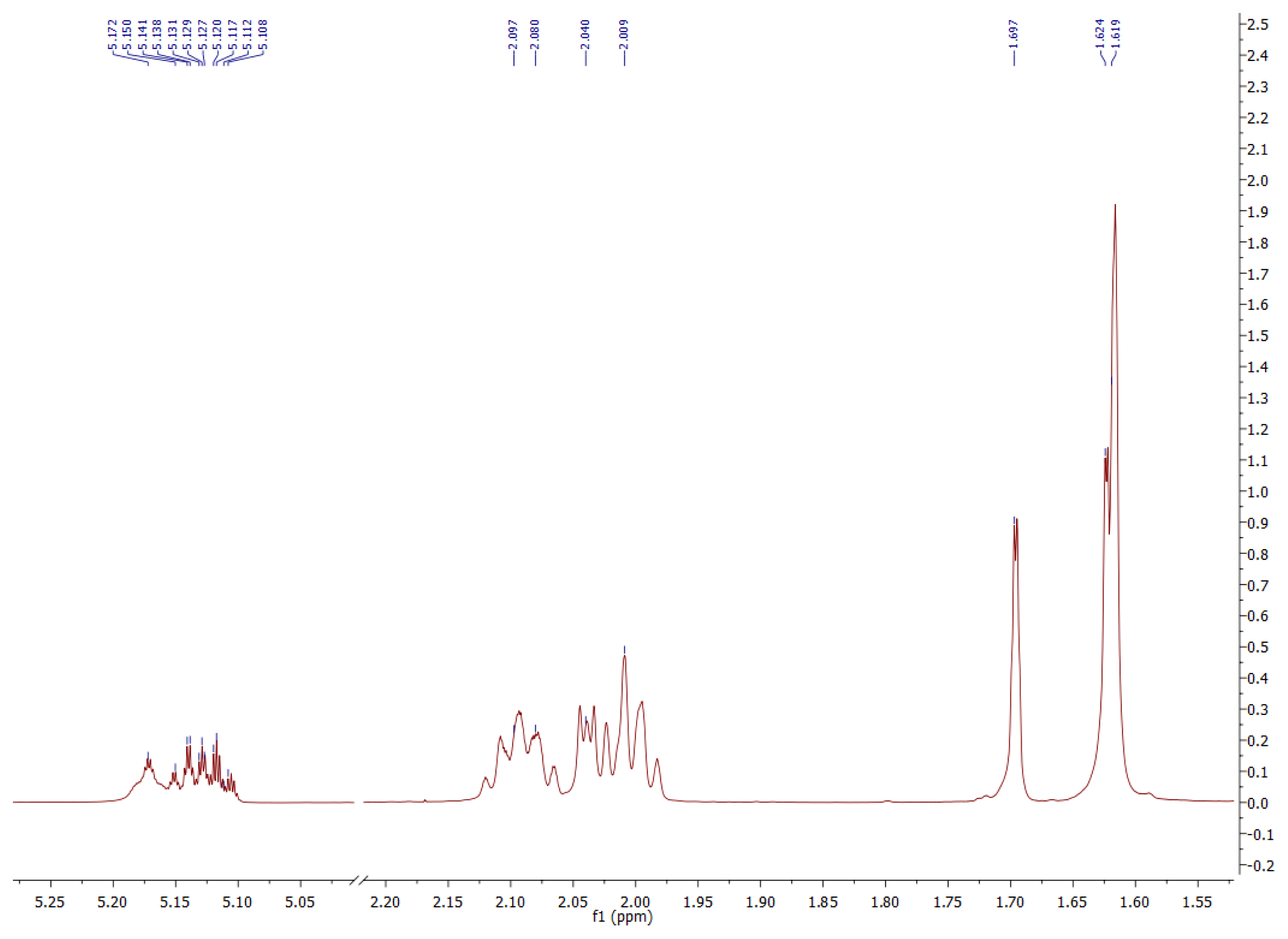

Supplement: Figure 7S — 1H NMR spectrum of squalene in CDCl3. [file turkjchem-47-1-294s7.tif]

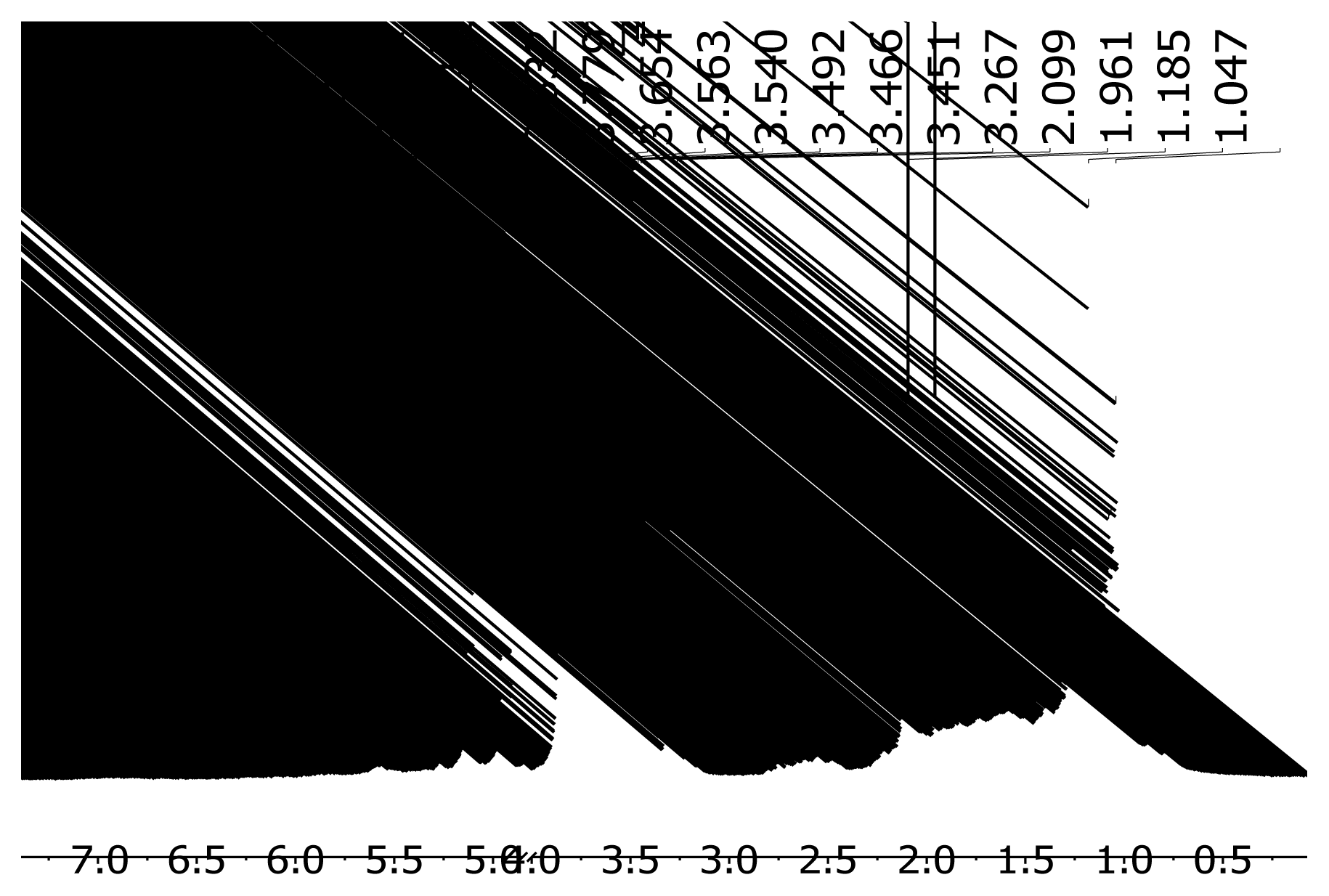

Supplement: Figure 8S — 1H NMR spectrum of squalene_Me-βCD complex in D2O. [file turkjchem-47-1-294s8.tif]

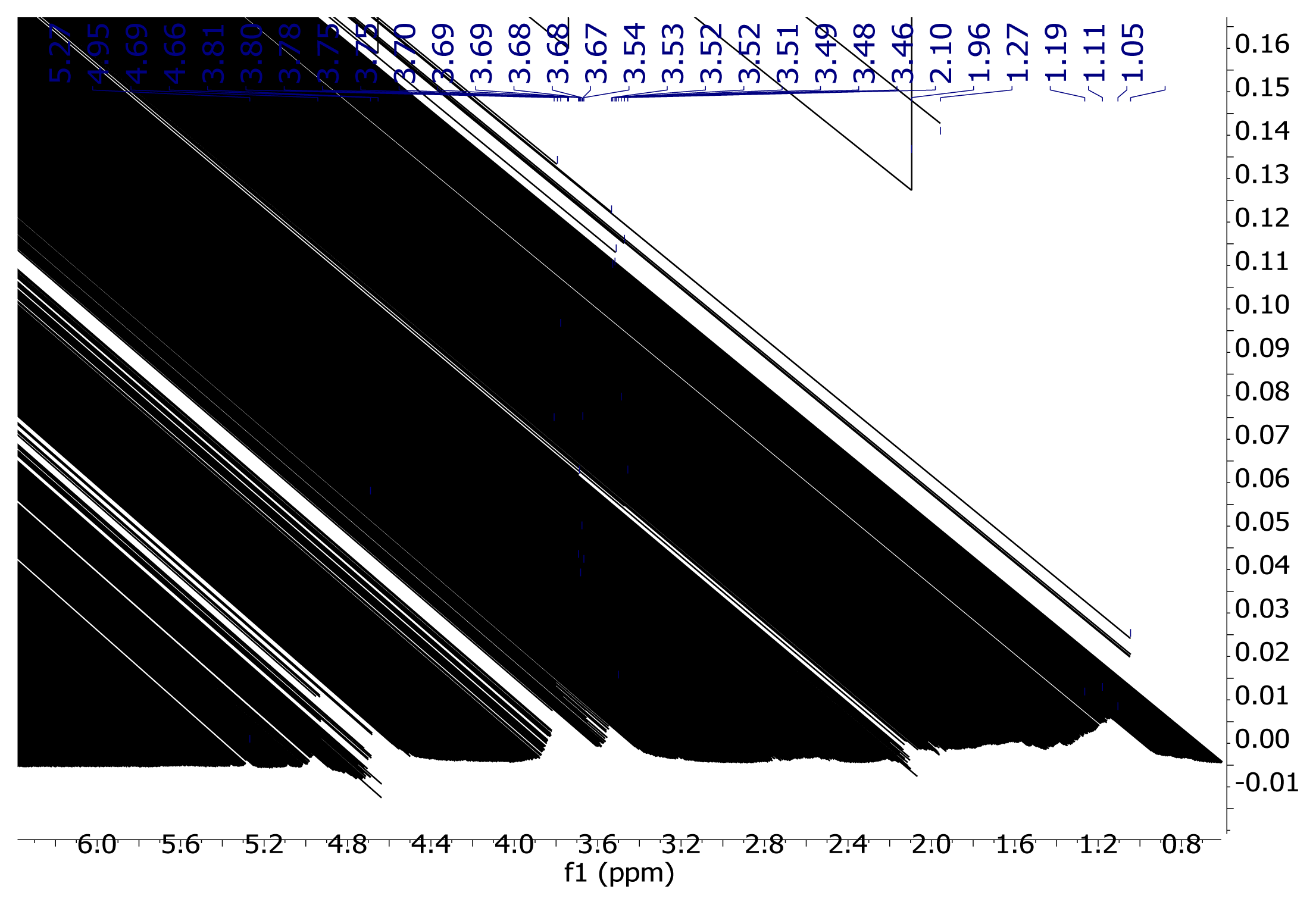

Supplement: Figure 9S — 1H NMR spectrum of squalene_ βCD complex in D2O. [file turkjchem-47-1-294s9.tif]

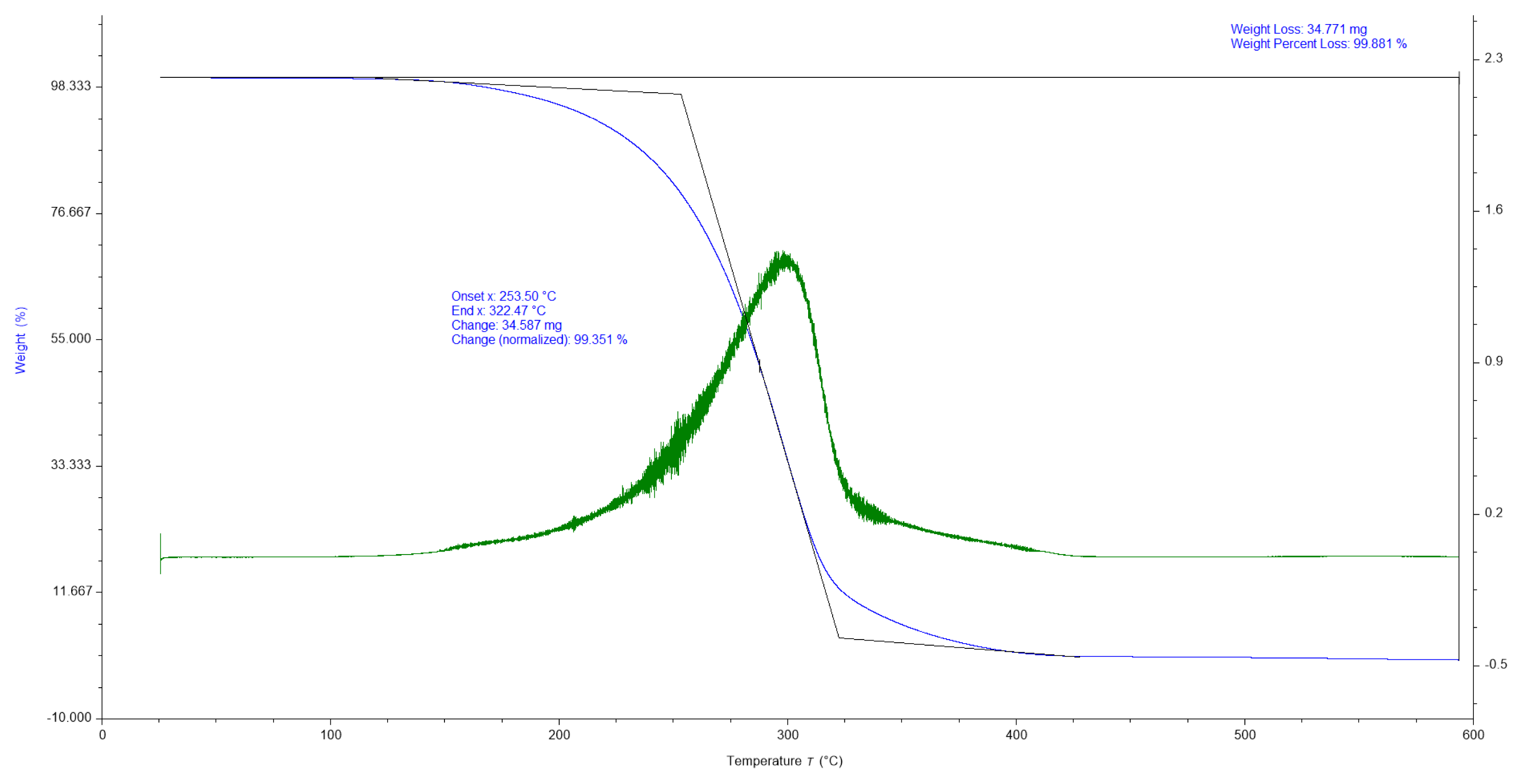

Supplement: Figure 10S — Thermogravimetric analysis of pure squalene. [file turkjchem-47-1-294s10.tif]
